# Political and intellectual legacy of Nikolai Ivanovich Astrov in Russia and in emigration

**DOI:** 10.3389/fsoc.2026.1763324

**Published:** 2026-03-27

**Authors:** Larisa Orchakova, Yuliya Smirnova, Elena Tokareva

**Affiliations:** Department of History, Moscow City University, Moscow, Russia

**Keywords:** archive, identity in exile, intellectual history, memory studies, Nikolai Astrov, political language, Russian liberal thought

## Abstract

**Introduction:**

This study aims to reconstruct the political and philosophical legacy of Nikolai I. Astrov, whose work bridges prerevolutionary liberalism and the intellectual traditions of the emigrant community, and to demonstrate how his writings transform the language of administrative service into a philosophy of memory, responsibility, and civic mission. The relevance of the research lies in the need to clarify how liberal ideas and categories of responsibility are reshaped under conditions of institutional collapse and forced displacement.

**Methods:**

The study is situated within an interdisciplinary framework that integrates tools from memory studies, intellectual history, and conceptual history. The corpus comprises 48 publications, 37 letters, one volume of memoirs, and six public lectures. Qualitative coding and lexico-semantic analysis were applied; Cohen’s *κ* coefficient reached 0.82, indicating strong inter-coder reliability.

**Results and discussion:**

The analysis suggests that Astrov articulated a distinctive model of civic identity grounded in the interplay between the ethics of action and archival reflection. The study contributes to scholarship on Russian liberal thought and émigré intellectual history by showing how administrative practice can be reconfigured as a language of ethical responsibility and memorial testimony. The practical value of the study lies in the potential application of its findings in courses on the history of political thought, public history, and the philosophy of memory, as well as in the development of digital cartographies of the intellectual networks of the Russian diaspora.

## 1 Introduction

Contemporary political and philosophical debates are marked by a crisis of liberal values, a degradation of the language of civic responsibility, and the growing dominance of authoritarian narratives in the public sphere. According to Freedom House, global freedom continues to decline: in 2024, only 20% of countries worldwide were classified as “free” in terms of political and civil rights, compared to 46% in 2005 (Freedom House, 2024). At the same time, pressure on the public sphere has intensified, affecting intellectual and migrant communities and reshaping the very notions of responsibility, memory, and political agency.

One of the most significant drivers of this transformation is the expanding scale of international migration. According to recent United Nations data, the global number of migrants has reached 281 million (UN, 2023). These processes are accompanied not only by shifts in sociocultural landscapes, but also by the emergence of new forms of transnational political engagement and resistance. Intellectual communities in exile are particularly sensitive to these changes, as memory of a disrupted homeland becomes not only an object of reflection but also a mode of political action (Chaudhary and Moss, 2019). In recent years, Russia has become a major source of outmigration among highly educated citizens. Over 2022–2023 alone, more than 800,000 people left the country (Centre for East-European and International Studies, 2023). Approximately 40% of those who departed continue their professional activities in education, science, and the media, forming intellectual diasporas that maintain connections to the Russian context while developing new practices of public expression abroad (Gaidar Institute, 2023).

Historically comparable dynamics can be observed in the experience of the Russian emigrant community of the early twentieth century. At that time, up to 2 million people found themselves outside Russia, establishing extensive networks of publications, archives, and educational institutions. In France and Germany alone, more than 200 Russian-language periodicals were in circulation, while the major emigrant archives eventually came to constitute up to 15% of the documentary holdings related to prerevolutionary Russian history (Online Archive of California, 2022). These parallels point to the value of drawing on historical experience to interpret contemporary challenges.

In this context, the figure of Nikolai I. Astrov acquires particular significance. In historiography, he has rarely been treated as an original thinker, with scholarly attention typically confined to biographical detail or administrative activity. Yet his writings articulate a conceptually coherent philosophical system in which the categories of responsibility, authority, identity, and mission are integrated across genres of public commentary, memoirs, and administrative documentation. Unlike the political rhetoric of his era, Astrov constructs a language of responsibility not through slogans but through institutional procedures and practices of accountability, making his approach especially relevant amid the contemporary crisis of trust in institutions (Linde, 2016).

This article presents an original and reflexive inquiry into how the liberal political lexicon can be reconstructed from understudied intellectual sources produced in exile. We not only reconstruct Astrov’s intellectual biography but also demonstrate how his texts function as instruments of normative philosophy. The analysis shows that archives and memoirs in his corpus operate not merely as repositories but as political practices, akin to contemporary forms of intellectual testimony under conditions of displacement.

The practical value of our study lies in the applicability of Astrov’s ideas to courses in intellectual history, migration studies, and archival theory. His conceptual framework offers tools for articulating a language of political responsibility under conditions of institutional uncertainty and transnational pressure. Theoretically, the study enriches the body of work on the intellectual history of Russian liberalism and expands the notion of public agency in contexts of migration and rupture. It also contributes to the development of interdisciplinary methodology by integrating political philosophy, the history of ideas, migration studies, and memory theory.

### 1.1 Literature review

Studies on memory politics in contemporary Russia reveal a shift from descriptive models toward an examination of the mechanisms through which the past is transformed into a resource of legitimation and mobilization. Current research focuses on how institutional practices (textbooks, museums, media) shape the language of responsibility and delineate the boundaries of permissible critique, thereby creating a foundation for reconstructing the intellectual trajectories of the early twentieth century and the interwar emigrant corpus (McGlynn, 2023; Soroka and Krawatzek, 2021). This shift illustrates how memory moves from the domain of preservation to that of political instrumentation. Within this logic, the ideas of N. I. Astrov demonstrate the transformation of administrative experience into a form of public ethics, in which visible action and accountability replace charismatic leadership (Kara-Murza, 2007).

Contemporary intellectual history integrates conceptual analysis with the study of knowledge infrastructures. Within this framework, it is shown that expert knowledge and political rationality form a shared space in which the boundaries of responsibility and the mechanisms of elite influence are negotiated (Rindzevičiūtė, 2020). This provides a basis for analyzing texts produced by thinkers who combined administrative practice with public writing, including Astrov, whose conceptual vocabulary emerged through institutions and archives. Studies on the history of the All-Russian Union of Cities and Moscow municipal governance describe Astrov’s position as “procedural liberalism” – an orientation toward legal mediation and accountability, opposed to charismatic leadership. This orientation links the ethics of responsibility with institutional rationality, which later becomes central to his emigrant identity (Shevyrin, 2003).

In international scholarship, the “civilizational turn” is viewed as an attempt to reinterpret identity by appealing to a distinctive historical trajectory and cultural codes (Linde, 2016). However, such approaches are rarely aligned with the liberal tradition of the early twentieth century, thereby risking the retrospective projection of post-Soviet meanings onto the prerevolutionary context. A comparison reveals a methodological gap between civilizational and liberal models of responsibility. Turning to Astrov’s corpus helps to partially bridge this divide and to demonstrate an alternative mode of political imagination grounded in administrative ethics rather than in the notion of cultural exceptionalism (Linde, 2016).

Recent scholarship on the Russian diaspora has advanced an approach that links intellectual history with the concept of political remittances. Emigrant communities are understood as sites of idea exchange in which norms and values circulate between countries of origin and settlement, shaping the political imagination of both contexts (Hartnett, 2020). For Astrov, this dynamic is evident in his transition from urban administration to archival and editorial initiatives, where publicity is generated through the procedures of preserving and describing sources. Sociological studies, in turn, highlight the constraints of transnational participation: external pressure and surveillance reduce willingness to engage in protest and foster practices of self-censorship (Almasalkhi, 2024; Moss, 2016). Within this context, Astrov’s archival work may be interpreted as a form of “slow protest,” a mode of civic expression that avoids overt confrontation.

Research on civic incorporation shows that migrants’ participation in movements and institutions reshapes their political imagination and their modes of engagement in the public sphere (Chow and Ren, 2023; Quinsaat, 2019; Zhao, 2021). This framework helps explain how intellectuals in exile construct audiences and legitimacy, redefining the notions of responsibility and mission. Astrov’s corpus demonstrates a shift from an administrative to a symbolic form of citizenship, in which text and memory become instruments of participation.

Studies of memory and autobiographical writing treat these practices as mechanisms for cultivating audiences and modeling civic virtue (Nathans, 2015). This perspective is productive for analyzing Astrov’s letters and memoirs, where autobiographical testimony functions as a means of ethical self-definition.

In comparative research on Russian and European philosophy, attention has been paid to the secularization of religious categories and the reinterpretation of concepts such as freedom and responsibility (Wilson, 2022). This allows Astrov’s corpus to be positioned alongside European traditions in which administrative accountability is understood as a practical expression of the ethics of duty. Classical texts of Russian religious and philosophical thought remain essential for analyzing the transformation of the concepts of responsibility and personal mission within the emigrant milieu. Studies of freedom, personhood, and communal responsibility trace how elements of this conceptual language are reinterpreted under new political conditions and incorporated into Astrov’s corpus as a “secular” adaptation of religious ethics (Berdyaev, 1937; Frank, 1987; Ilyin, 2018).

The historiography on Astrov remains fragmented and focuses primarily on biographical details and institutional roles, without engaging in conceptual analysis of his writings (Shevyrin, 2003). Scholarly interest in him has been largely confined to reference works on Russian liberalism, a limitation that reflects the dominance of approaches treating liberalism as a moral or national ideology. Yet Astrov’s texts make it possible to approach liberal ethics as an institutional practice expressed through documents, reporting procedures, and archival work. This dimension is rarely addressed in studies centered on religious philosophers or late-Soviet memoir literature (Hartnett, 2020; Nathans, 2015; Soroka and Krawatzek, 2021).

Although Astrov’s primary texts constitute a substantial source base, they have remained almost entirely unexamined in academic discourse. Despite the existence of an extensive corpus of letters, reports, and lectures, these materials have scarcely been incorporated into scholarly circulation and are mentioned only sporadically, without systematic analysis of their conceptual apparatus (Report of the RZGK [app. 1, p. 87], 1921; Report of the RZGK [app. 1, p. 89], 1922). Moreover, Astrov’s figure is practically absent from contemporary intellectual history, where attention tends to focus on more canonized emigrant authors, exacerbating the gap between the available source base and its theoretical interpretation (Clowes, 2011).

Bridging this gap requires engaging with Astrov’s corpus as an independent intellectual resource that integrates administrative practice with public history. His writings illustrate how procedural responsibility transforms into a civic ethic in which the archive becomes an instrument of legitimizing and preserving collective memory (Etkind, 2013; Miller and Lipman, 2012).

A methodological gap persists between structural approaches, which conceptualize memory as an institutional practice, and hermeneutic interpretations, which analyze the subjective experience of testimony. Examining Astrov’s texts bridges these frameworks by demonstrating how the liberal idea persists outside the state, transforming into a form of knowledge and an ethic of participation (Kara-Murza, 2007; Linde, 2016; Rodionova, 2015). From this perspective, Astrov emerges not only as an administrator and publicist but also as a thinker, linking Russian and diasporic traditions and revealing the mechanisms by which the liberal language of responsibility maintains its continuity.

### 1.2 Problem statement

In historiography, N. I. Astrov is predominantly presented as a liberal politician and organizer of archival work, while his writings are rarely examined as a coherent philosophical project. An interdisciplinary study grounded in primary archival materials and the methodological tools of memory studies is required to place Astrov at the center of discussions on Russian liberal thought. Reconstructing Astrov’s conceptual vocabulary will clarify the interrelations among power, responsibility, and identity, which are essential for advancing research on political language and memory. The relevance of turning to his legacy is further reinforced by the contemporary crisis of liberal values and the search for new foundations of civic responsibility.

The aim of this study is to reconstruct and analyze the political and intellectual legacy of N. I. Astrov as an independent thinker and to identify his contribution to shaping the discourse of responsibility, authority, identity, and historical mission in both the prerevolutionary and emigrant periods.

## 2 Objectives

1. To assemble a source base consisting of Astrov’s publications in “Russkaya mysl’” and “Vozrozhdenie,” the book “Memoirs,” his corpus of letters and reports to the RZGK, and contemporaries’ memoirs held in the RGALI and RGB collections.
2. To conduct a conceptual reconstruction of Astrov’s key categories and trace their evolution across historical periods.
3. To apply the analytical tools of memory studies to examine genres, addressees, and modes of testimony.
4. To carry out a comparative analysis with European liberal thinkers and Astrov’s Russian contemporaries, identifying points of convergence and divergence.
5. To correlate the findings with current international scholarship and determine Astrov’s place within an alternative trajectory of Russian political thought.

## 3 Methodology

The methodological framework of this study is built upon the integration of three approaches that together enable the connection of textual reconstruction with institutional and reception analysis. First, the perspective of memory studies is employed, treating memory as a set of practices of selection and forgetting, and as an ensemble of mediating structures through which intellectual texts acquire audiences and normative authority. This component draws on the concepts of cultural memory and memory carriers, as well as on observations concerning contemporary regimes of public history that establish the rules for permissible citation of the past and the languages of responsibility in the present (Assmann, 2011; Erll, 2011; McGlynn, 2023).

Second, the study relies on the tools of intellectual history and conceptual history. The text is treated as an act situated within a specific institutional context, and categories are analyzed in terms of their evolving usages and functions. Theoretical reference points include the contextual approach to political language, which enables linking semantic shifts to changes in social addressees and generic forms (van den Elzen, 2024).

Third, the reconstructed categories are correlated with political-philosophical debates on action, guilt, and responsibility. These approaches allow for linking individual texts with collective practices of knowledge production and memory formation. This comparative horizon structures the discussion of the relationship between normative duty and administrative practice and underscores the distinction between moral evaluation and legal status – an essential condition for the accurate interpretation of sources from the prerevolutionary and emigrant periods (Arendt, 1958; Jaspers, 1947; Rindzevičiūtė, 2020).

### 3.1 Study design

The object of this study is the corpus of texts authored by N. I. Astrov and his immediate intellectual circle. This corpus includes journal articles and short pieces published in “Russkaya mysl’” and “Vozrozhdenie,” the book “Memoirs,” epistolary materials, as well as reports prepared in connection with the work of the Russian Historical Committee Abroad (RZGK). To ensure reproducibility, two temporal segments are delineated. The first covers the years 1905–1917; the second spans 1918–1942. Based on catalogues and archival inventories, 48 publications and short pieces, one volume of “Memoirs,” 37d letters and administrative notes on urban governance, and six reports related to the RZGK were identified and included (Appendix 1). Archival units and copies were obtained from RGALI and RGB. For RZGK materials, we used archival reference files and copies of printed editions preserved in emigrant publications and private collections. All texts were digitized with ABBYY FineReader and then manually proofread. Dictionaries from the early twentieth century were employed for work with prereform orthography.

Comparative materials were selected according to the principle of thematic and generic commensurability. Within the European context, the study incorporates Hannah Arendt’s writings on public action and the conditions of political space, as well as Karl Jaspers’s works on the forms of guilt and the limits of collective responsibility. Isaiah Berlin’s analyses of freedom and political judgment and Raymond Aron’s reflections on the relationship between expertise and democracy in modern politics are also utilized. On the Russian side, the texts of Petr Struve and Vasily Maklakov serve as key points of reference within the liberal lexicon, while Georgii Fedotov’s works on the Christian ethics of public service provide an additional conceptual anchor. For genre and reception analysis, memoirs by Alexander Kizevetter and George Vernadsky are used to reconstruct norms of service as well as academic and municipal culture, which form an important contextual frame for interpreting Astrov’s writings (Antoshchenko and Ponomarev, 2024; Arendt, 1958; Aron, 1965; Berlin, 1958; Fedotov, 1991; Jaspers, 1947; Kizevetter, 1997; Maklakov, 2001; Struve, 2004).

Inclusion criteria were established in advance to ensure accurate reconstruction of the corpus. We included texts with confirmed authorship by Astrov or with editorial attribution. Short chronicle notes lacking conceptual substance and materials in which Astrov appeared only in the third person were excluded. For the comparative corpus, genre correspondence and audience comparability were strictly observed to minimize distortions arising from differences in media formats.

### 3.2 Data analysis

The analytical procedure integrates qualitative coding with quantitative components. Annotation was conducted in NVivo 14 using a hierarchical coding tree structured around four cluster categories. To ensure procedural robustness, two independent coders worked on a 20% subcorpus. Intercoder agreement was assessed using Cohen’s kappa, yielding a score of 0.82, which indicates high consistency. All discrepancies were discussed until consensus was reached, and they were subsequently incorporated into the finalized coding scheme. For lexico-semantic processing, MyStem and UDPipe were employed to support lemmatization and normalization with attention to prereform orthography. Concordances and collocations were generated in AntConc. These procedures do not replace qualitative reading; rather, they serve to verify the stability of observations and provide a foundation for frequency tables and relational visualizations.

The comparative analysis was conducted on two levels. The conceptual level juxtaposes interpretations of responsibility, guilt, and mission in Astrov’s writings and those of European authors, accounting for differences in genre and intended audiences. The historical-contextual level compares Astrov with his Russian contemporaries – primarily Struve, Maklakov, and Fedotov – making it possible to distinguish unique and shared features of the liberal lexicon and to trace the evolution of concepts under the institutional ruptures of the early twentieth century and the emigrant period. To control for potential distortions, reflexive validation was applied: approximately 15 % of the corpus was reread, with all interpretive decisions documented in a research log. The role of the researcher was conceptualized as participatory, which helped minimize subjective drift in the interpretation of key concepts.

### 3.3 Ethical issues

Ethical considerations pertain to the use of historical materials. For unpublished letters and administrative documents, all archival regulations were observed, including requirements for citing collection, inventory, and file numbers. No personal data belonging to living individuals is included in the publication.

### 3.4 Limitations of the study

The limitations of the study stem from the incompleteness of prerevolutionary and emigrant periodicals and from the fragmentary nature of the epistolary materials. Normalization of prereform orthography may affect automatic lemmatization; this was mitigated through double manual proofreading and verification of keyword contexts. Differences in target audiences across the comparative corpus may distort frequency profiles. This risk was reduced by selecting comparable genres and documenting all media parameters in the coding protocol.

## 4 Results

The contribution of this study lies in the combined use of quantitative and qualitative analysis of N. I. Astrov’s textual corpus, enabling a systematic reconstruction of his writings as a coherent philosophical project integrating the categories of responsibility, authority, memory, and identity, rather than viewing him solely as an administrator or public commentator. The innovative aspect of the approach consists of applying tools of lexico-semantic reconstruction alongside methods from memory studies, which enabled an empirical demonstration of the shift from an administrative-legal to an ethical-existential language within liberal thought. This study thus opens a new perspective on the Russian emigrant experience, conceptualizing it not only as a sphere of political adaptation but also as a context for philosophical rearticulation of duty, authority, and historical mission through archival and memorial practices.

The analysis of Astrov’s corpus, organized into two chronological periods (1905–1917 and 1918–1942), revealed both the evolution of his views and the structural characteristics of his conceptual apparatus. This periodization should be understood as an analytical reconstruction of discursive transformations rather than as a normative evaluation of institutional effectiveness or historical rupture. It is employed heuristically in order to trace shifts in conceptual emphasis and modes of articulation across different political contexts. Qualitative coding followed by quantitative analysis confirmed the hypothesis that Astrov should be regarded as an independent thinker whose intellectual legacy extends beyond administrative or memoir literature.

For Astrov, the period 1905–1917 is marked by a language of institutional responsibility directed toward municipal voters, the bureaucracy, and the Duma-oriented readership of “Russkaya mysl.” In the late-imperial political environment – characterized by limited constitutional mechanisms and wartime mobilization practices – authority is understood as order and competence legitimized through procedure and accountability. Responsibility appears as a juridical-administrative obligation to the urban community, with its center of gravity located in budgets, audits, and regulations. Publicity functions as a tool of oversight and visible action through open meetings and the publication of reports. During this period, archives and memory serve as infrastructures of administrative record-keeping, ensuring the verifiability of decisions. Identity and mission remain secondary and are interpreted through the ethics of public service and the civic education embedded in municipal self-government.

After 1917, within the emigrant environment of the Russian diaspora and the archival networks of Prague, the addressee, genres, and logic of public action undergo a profound transformation. The loss of former institutions and the external constraints on public political activity shift the center of gravity toward transnational channels of communication. Mission becomes the moral program of the diaspora, substituting for institutional objectives. Identity is reconstructed as a professional-civic role – that of witness, custodian, and curator of memory – formed through practices of solidarity. Memory and the archive become modes of testimony and vehicles for the public representation of responsibility. Responsibility itself moves into an ethical-existential register, articulated as a duty toward the past and the future of the intended audience. Authority becomes decentralized and is reinterpreted as a form of legitimacy arising from procedures of testimony and from archival-editorial work. Publicity is achieved through infrastructures of memory – collections, inventories, catalogues, and publishing platforms – rather than through assembly halls.

Taken together, these changes indicate a shift from accountability to testimony. Before 1917, authority rested on regulation and competence; responsibility was reduced to administrative reporting; publicity emerged through controlled open procedures; archive and memory functioned as instruments of record-keeping and verification; identity was defined by the role of public servant; and mission corresponded to civic education and the tasks of municipal self-government. After 1917, authority derives from the networks of memory institutions; responsibility acquires the meaning of an ethical-existential obligation; publicity becomes a form of archival-editorial representation; archive and memory gain the status of testimony and foundations of collective identity; identity becomes the core of service within the diaspora; and mission turns into the semantic framework of communal survival.

A frequency analysis of key political-philosophical categories in Astrov’s texts confirms a distinct conceptual shift after 1917. Whereas in the prerevolutionary period the dominant concepts were “authority” and “responsibility,” closely tied to the specifics of urban administration, in the emigrant period, “mission” and “identity” come to the forefront, reflecting the search for new foundations of collective existence within the diaspora. This shift in emphasis is clearly illustrated in Table 1.

**Table 1:** Evolution of the frequency of key categories in N. I. Astrov’s text corpus (in normalized units per 10,000 words).

Category	Period 1905–1917	Period 1918–1942	Dynamics
Responsibility	24.5	28.7	↗
Power	31.2	18.9	↘
Identity	8.4	22.1	↗
Mission	12.1	35.6	↗
Publicity	15.8	14.2	→
Archive	6.3	19.4	↗
Memory	9.7	25.3	↗

Source: author’s compilation.

As the table illustrates, concepts such as “archive” and “memory” show the most substantial growth, fully corresponding to Astrov’s shift from a practitioner of municipal administration to a custodian and interpreter of historical experience in exile. At the same time, the stable frequency of the category “publicity” indicates its transversal character, functioning as a connective element between his prerevolutionary administrative practice and his emigrant public writing.

**Figure 1:**
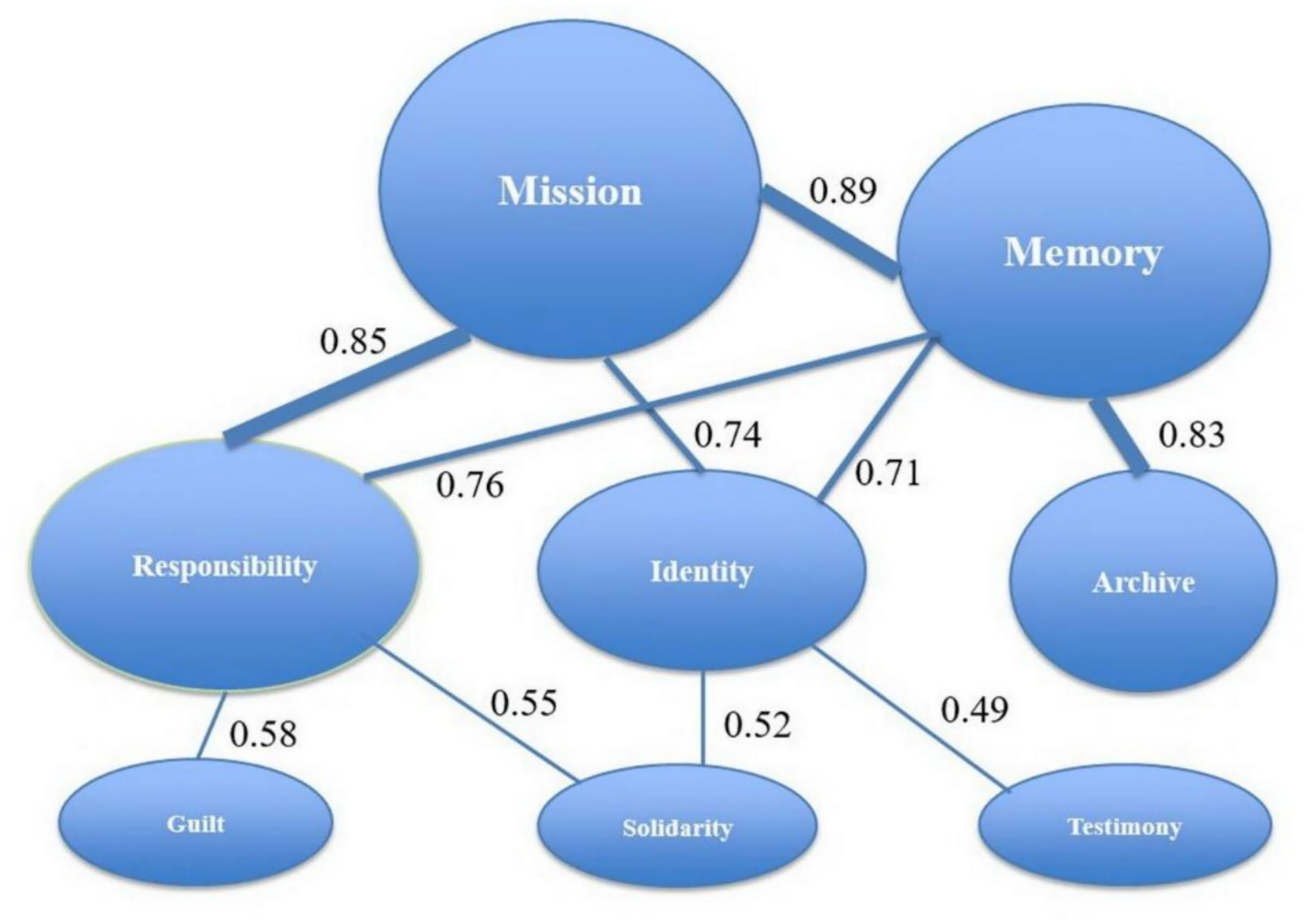
Semantic network of key concepts in the emigrant period (1918–1942) based on collocation analysis. Source: author’s compilation.

To gain a deeper understanding of the structure of his discourse after emigration, a collocation analysis was applied to identify semantic associations among key concepts. The reconstructed semantic network, visualized in Figure 1, reveals a clear hierarchy organized around two central nodes: “Mission” and “Memory.” The strength of the link between these concepts (collocation index 0.89) indicates their inseparable unity in Astrov’s thought: the historical mission of the Russian emigrant community is understood primarily as the mission of preserving and transmitting memory, while memory itself acquires tangible form through archival work. Equally significant is the stable association between “Mission” and “Responsibility” (0.85), which in the context of exile loses its strictly administrative-legal meaning and becomes imbued with the existential sense of duty toward the past and the future.

Peripheral but conceptually important associations reveal the ethical-philosophical transformation of Astrov’s language. The statistically significant link between “Responsibility” and “Guilt” (0.58) marks the reflexive dimension of his thought, in which responsibility includes the work of interpreting collective guilt and historical failure. The associations between “Identity” and “Solidarity” (0.52) and “Testimony” (0.49) show that the new identity in the diaspora is constructed not through ethnic or state markers, but through practices of mutual support and acts of public testimony about the past.

Taken together, the quantitative data demonstrate convincingly that Astrov’s emigrant discourse constitutes a coherent system in which political-philosophical categories (“authority,” “responsibility”) are transformed into existential and memorial categories, forming a distinctive language of historical experience and moral duty centered on “Mission” and “Memory.”

The evolution of Astrov’s ideas becomes particularly clear when the genre-specific features of his sources are compared. An analysis of his publications in “Russkaya mysl’” shows that his texts were oriented toward shaping a language of civic consciousness within the imperial public sphere. During this period, his reflections on responsibility are institutional in nature, embedded in debates on the distribution of powers between the city and the state. By contrast, his emigrant writings in “Vozrozhdenie” and his reports for the Russian Historical Committee Abroad function as acts of testimony aimed at constituting a new, diaspora-based audience. For him, the genres of memoirs and historical reports become not merely a means of recording the past but a tool for constructing normative models of virtue and service, consistent with the methodological premises of memory studies. This shift from an administrative language to one of existential and collective responsibility is reflected in Table 2, which systematizes the main conceptual pairs across the two periods.

**Table 2:** Evolution of conceptual relations in the discourse of N. I. Astrov.

Period	Dominant conceptual pairs	Context and audience
1905–1917	Responsibility – Competence; Authority – Law; Publicity – Oversight	Institutional, within the imperial public sphere
1918–1942	Responsibility – Guilt; Mission – Solidarity; Memory – Archive; Identity – Service	Existential-ethical, addressed to a diaspora audience

Source: author’s compilation.

Thus, the analysis demonstrates that Astrov’s intellectual project constitutes a coherent system that evolved from a liberal philosophy of municipal self-government into a distinctive philosophy of exile, in which the categories of historical mission, collective memory, and ethical responsibility merge into a unified discourse aimed at preserving identity and moral authority under conditions of lost political subjecthood.

An examination of Nikolai Astrov’s political and philosophical legacy makes it possible to view him not only as a figure of the Russian emigrant community but also as an original thinker who reflected on the nature of responsibility, identity, and authority amid historical catastrophe. The comparison that follows is intended as an analytical and thematic juxtaposition rather than a claim of direct intellectual influence; it serves to situate Astrov’s conceptual vocabulary within a broader field of twentieth-century reflections on responsibility, authority, identity, and public action. These themes resonate with the intellectual concerns of European liberals – in particular Hannah Arendt, Karl Jaspers, and Isaiah Berlin – who likewise contemplated the moral foundations of politics in times of crisis (Arendt, 1958; Berlin, 1958; Jaspers, 1947).

The category of responsibility occupies a central place in Astrov’s writings. In an early report for the Moscow Municipal Administration (1912), he wrote: “Authority begins with accountability. Where there is no recognized obligation to answer, arbitrariness begins.” This formulation brings him close to Jaspers’s conception of “moral guilt” as a precondition for genuine political action (Jaspers, 1947). In the postwar emigrant period, Astrov came to describe the archive not only as a means of preservation but also as an instrument of testimony. In a 1934 letter to S. V. Kizevetter, he emphasized: “The archive is a form of guilt transferred onto paper” (App. 1, 63). Like Arendt, he understood memory not as the mere storage of the past but as an act requiring both personal and collective commitment (Arendt, 1963). In this sense, Astrov aligns with both Arendt and Jaspers in seeking to transform historical knowledge into a moral and political stance.

Another essential node in Astrov’s philosophy is publicity as a condition of responsibility. In his articles in “Vozrozhdenie” (1930s), he repeatedly returns to the theme of “visible action,” arguing that the emigrant experience transforms the archivist into “a witness before a silent future” (App. 1, 29; 30; 32; 37). Here, he converges with Arendt’s idea of action as a mode of being among others, while the notion of the public sphere in Astrov’s work acquires archival and institutional dimensions. Whereas Arendt emphasizes the spontaneity of action, Astrov speaks of the duty to preserve the contours of the public sphere even under conditions of institutional collapse (Arendt, 1958).

Identity, as evident from Astrov’s memoirs, is understood not as a stable attribute but as the outcome of “service experience,” where professional competence, memory of a destroyed homeland, and adaptation to new political regimes intersect. This position aligns with Isaiah Berlin’s view of identity as a tension between individual choice and cultural context (Berlin, 1958). Unlike Berlin, however, Astrov emphasizes not pluralism but discipline: identity for him arises as a form of obligation, inseparable from the labor of memory and archival inventory.

In his reflections on authority, Astrov gravitates toward an administrative and legal tradition. In the articles he wrote during his work at the Moscow Municipal Administration, he spoke of “authority arising from order and accountable to it” (App. 1, 50; 58; 64). This position aligns more closely with Tocqueville than with Arendt. Whereas Arendt understands authority as emerging from collective action and consensus (Arendt, 1958), Astrov conceives it as the result of service, competence, and accountability. Such an approach implies a more bureaucratic yet more stable conception of authority, comparable to the French administrative tradition elaborated by Aron (1955). A more detailed comparison of the key conceptual categories is presented in Table 3.

**Table 3:** Comparison of key categories in N. I. Astrov and western thinkers.

Category	Responsibility	Authority	Identity	Public action
N. I. Astrov	Authority begins with accountability. Where there is no recognized obligation to answer, arbitrariness begins. Responsibility is a legal and moral obligation to society.	Authority as institutional order and competence: “authority arising from order and accountable to it.”	Identity as “service experience” and as memory of a destroyed homeland.	“An archive is a form of guilt transferred onto paper.” Testimony as a form of action and as a duty of memory.
Hannah Arendt	Responsibility is the awareness that no one can act in our place. Personal and collective responsibility emerges through action.	Authority arises where people act together.	Identity is generated through public action and through recognition by others.	Action is a form of presence in the world that gives meaning to human life.
Karl Jaspers	Everyone bears guilt for allowing evil to occur. Moral responsibility is a condition of freedom.	Authority is an expression of communication and mutual understanding between people.	Identity is the result of self-understanding at the limits of existence.	Action is the realization of authentic existence through dialogue.
Isaiah Berlin	Responsibility is the result of individual choice amid conflicts of values.	Authority is the limitation of arbitrariness for the preservation of freedom.	Identity is the space of choice between competing values.	Action is a moral decision taken under conditions of constraint.
Raymond Aron	Responsibility is the rational discipline of the citizen, restraining ideology.	Authority is a form of political reason rather than coercion.	Identity is rational self-placement within society.	Public action is participation in democratic reason.

Source: author’s compilation.

In summary, several points of convergence and divergence can be identified. Like Arendt, Astrov emphasizes the necessity of action grounded in memory. Yet whereas Arendt understands such action as a breakthrough toward the new, Astrov views it as a duty to restore disrupted institutional connections. He is closer to Jasper’s in his interpretation of moral responsibility as the foundation of political existence. His concept of identity is built on labor, archive, and order, rather than on the more hermeneutic models of Berlin and Arendt. In his understanding of authority, he aligns more closely with Tocqueville and Aron, both of whom emphasize legal mediation and administrative legitimacy.

Russian liberal thought of the early twentieth century, in turn, provided Astrov with an intellectual context in which the categories of responsibility, authority, and service carried not only administrative but also moral significance. In his essay “Great Russia,” Petr Struve regarded statehood as a form of national self-awareness and linked freedom with order and legality. He believed that civic discipline and responsibility make the development of a constitutional state possible (Struve, 2004). This understanding brings him close to Astrov, for whom municipal self-government and archival culture constituted practices of rational responsibility. The difference between them lies in scale: Struve prioritizes the national-historical level, whereas Astrov emphasizes the institutional-administrative. Struve wrote about the formation of the nation as a moral-political subject, while Astrov associated civic consciousness with everyday forms of administrative duty and record-keeping.

The closest parallels to Astrov are found in the ideas of Vasily Maklakov and Georgii Fedotov. Reflecting in his memoirs on the work of the First and Second State Dumas, Maklakov understood authority as a process of public mediation and responsibility (Maklakov, 2001, 2023). His conception of politics as legal dialogue corresponds to Astrov’s ideal of the public servant accountable to society. Georgii Fedotov developed the idea of ethical freedom, combining personal conscience with public mission. In his writings of the late 1930s, he argued that democracy requires not only institutions but also the cultivation of the heart (Fedotov, 1991; Lyuks, 2015). This understanding of responsibility as spiritual service and duty toward history brings him close to Astrov, for whom archive and testimony are expressions of moral obligation to the past. Thus, Struve represents the rational-state dimension of liberalism, Maklakov embodies its legal practice, and Fedotov articulates its spiritual side. Astrov unites these approaches through the experience of municipal responsibility and a culture of memory, joining liberal ethics with the practice of archival action.

## 5 Discussion

Our study confirms that N. I. Astrov evolved from a focus on municipal authority and responsibility to a reflection on historical mission and identity in the context of emigration. This conceptual shift aligns with the “civilizational turn” noted in the literature on Russian discourse (Linde, 2016). International scholarship shows that historical memory has become a political resource in contemporary Russia, where struggles over the past serve as tools of legitimation (Soroka and Krawatzek, 2021). Astrov’s work in preserving archives abroad anticipates this tendency. He constructed an independent infrastructure of memory outside the control of the Soviet state, advancing a liberal interpretation of responsibility. His autobiographical texts not only record events but also set a normative example of civic responsibility for the emigrant community, corresponding to the view that memoirs create an audience and establish norms of virtue (Nathans, 2015).

At the same time, some findings in international research diverge from our observations. Studies on transnational activism emphasize the severe constraints faced by diasporas (Chaudhary and Moss, 2019). Astrov’s experience presents a more complex picture: despite the absence of state support, he maintained intellectual and institutional activity for decades, challenging the assumption that emigration inevitably leads to political passivity. Unlike contemporary regimes that aim to influence diasporas (Wackenhut and Orjuela, 2023), the Soviet state completely excluded the liberal emigrant community, enabling Astrov to develop his ideas autonomously and to shape an alternative trajectory of political thought. His example challenges the notion that, without a “metropole,” the voice of an intellectual in exile must inevitably fall silent.

Astrov also fits within global patterns of political engagement among migrants. His sustained interest in Russia’s fate corresponds to findings that prior political experience increases engagement in homeland affairs (Soehl et al., 2024). Many emigrants inhabit two civic contexts simultaneously (Chow and Ren, 2023), yet Astrov remained oriented toward the Russian public sphere. Unlike, for example, Chinese dissidents whose integration into the political movements of host countries is often constrained (Zhao, 2021), Astrov devoted himself to emigrant organizations and publications, consolidating fellow Russians around the ideas of responsibility and mission. Thus, even outside his homeland, he acted as a transnational intellectual, continuing a dialogue with the domestic tradition. A comparable phenomenon is described in the case of the emigrant philosopher Alexandre Kojève, who, in France, reinterpreted the ideas of Vladimir Solovyov while maintaining intellectual connections to Russian philosophy (Wilson, 2022).

Our study demonstrates that N. I. Astrov’s intellectual legacy extends beyond his biography and administrative career. It is manifested in the way his language of responsibility and public service became part of a broader tradition in Russian political thought. The concept of responsibility as a moral duty and publicity as a form of civic ethics continues in the writings of Georgii Fedotov, for whom freedom is inseparable from the acknowledgment of duty toward the other (Fedotov, 1946). Through this line, Astrov becomes part of the continuum of religious-liberal philosophy, where service is understood as a condition of genuine citizenship. His rethinking of the role of the archive as a space of action and of the morality of memory influenced the subsequent development of humanities scholarship on public history and memory politics in Russia (Etkind, 2013; Miller and Lipman, 2012).

The significance of Astrov for the intellectual tradition lies in his ability to connect two levels of analysis – the normative and the institutional. He demonstrated that liberal ethics can exist not only in theoretical texts but also within the infrastructure of memory: in the organization of archives, publishing series, and the formation of collective practices of responsibility. These ideas anticipated contemporary understandings of memory as a moral resource and help explain why, in post-Soviet humanitarian policy, the archive has become central as a site of public testimony and repentance (Memorial, 2025; Oustinova-Stjepanovic, 2023).

Thus, Astrov’s legacy links two layers of the Russian political tradition: the liberal culture of self-government and the emigrant philosophy of responsibility. He shows how ideas of civic virtue and duty can persist and evolve beyond the nation-state framework. His intellectual trajectory bridges early Russian liberalism and contemporary public history, enabling us to view Astrov as an important precursor of what can now be described as the humanistic dimension of memory politics (Etkind, 2013; Fedotov, 1946; Miller and Lipman, 2012; Oustinova-Stjepanovic, 2023).

The interdisciplinary approach used in this study – at the intersection of intellectual history, migration sociology, and memory studies – enabled the examination of Astrov as a key figure within a network of transnational memory and knowledge (Rindzevičiūtė, 2020). The application of the concept of a civilizational political language (Linde, 2016) helps explain the shift in his rhetoric toward articulating a special mission for Russia, while the analysis of memoirs illuminated how ethical norms are established through narrative. The reliability of the findings is supported by a broad source base and data verification: despite the incompleteness of certain archives, the combination of quantitative and qualitative textual analysis confirmed the identified patterns, strengthening the credibility of the results.

The findings allow for a more precise understanding of Astrov’s relevance to contemporary political thought. His legacy demonstrates that the liberal tradition can maintain coherence even outside the nation-state, relying not on institutions of power but on practices of memory, civic responsibility, and a culture of public service. For modern liberalism, this example is important as a reminder of the connection between freedom and duty, and of the possibility of transparency and accountability even under conditions of political exile. In the Russian context, Astrov’s ideas help to reinterpret liberalism not as an elite ideology but as an ethic of participation and responsibility toward society. For emigrant intellectual life, his example shows that intellectual autonomy can function as a form of resistance to forgetting and as a means of preserving cultural continuity. More broadly, his concept of the “archive as a form of guilt” anticipates the contemporary understanding of memory as a moral resource underpinning public history and the politics of recognition. Thus, Astrov’s legacy remains relevant not only for studying the past but also for analyzing how liberal values can survive and evolve amid institutional loss and ideological pressure.

## 6 Conclusion

In the course of this study, a multidisciplinary analysis of Nikolai Astrov’s political and philosophical legacy was carried out, based on a corpus of 48 publications, 37 letters and notes, one volume of memoirs, and six reports prepared for the Russian Historical Committee Abroad. This approach enabled the identification of four central categories in Astrov’s work: “responsibility,” “authority,” “mission,” and “identity.” The analysis revealed a shift from juridical-administrative to ethical-existential meanings during the emigrant period and documented stable intellectual parallels with Arendt, Jaspers, and Fedotov. This study offers a systematic reconstruction of Astrov’s writings, presenting him not only as an archivist and liberal administrator but as a thinker whose work articulates a distinctive conception of political responsibility, archival memory, and service-based identity. Comparison with Russian and Western authors helped to establish the uniqueness of his approach and to situate him within the field of intellectual history.

### 6.1 Practical implications and further research

The results of this study are applicable in academic courses on the history of political thought, migration studies, memory studies, and the history of archival practice. The materials may also be used in public history, museum pedagogy, and in the rethinking of archives as institutions of testimony.

In future research, it will be necessary to expand the source base through international archival collections, apply digital mapping to reconstruct the intellectual networks of the Russian emigrant community, and supplement the corpus with memoiristic and publicistic texts produced by the second generation.

## Data Availability

The original contributions presented in the study are included in the article/Supplementary material, further inquiries can be directed to the corresponding author.
