# Supplementary material for: Political and intellectual legacy of Nikolai Ivanovich Astrov in Russia and in emigration
Source: Front Sociol. 2026 Mar 27;11:1763324. doi: 10.3389/fsoc.2026.1763324 (PMC13065707; doi:10.3389/fsoc.2026.1763324)
Supplement: Supplementary file 1 [file Supplementary_file_1.docx]

**Appendix 1**

A. Publications and Notes in Periodicals – 48 entries

Format: No. | Year | Publication | Title | Issue and pages

1 | 1906 | Russkaia mysl’ | Municipal Self-Government as a School of Civic Consciousness | No. 3, pp. 45–63 | RGALI, f. 941, op. 10, d. 123, fols. 5–18

2 | 1906 | Russkaia mysl’ | Urban Representation and the Responsibility of the City Duma | No. 7, pp. 12–28 | RGALI, f. 941, op. 10, d. 124, fols. 1–14

3 | 1907 | Russkaia mysl’ | On the Limits of Municipal Administrative Authority | No. 1, pp. 79–98 | RGALI, f. 941, op. 11, d. 18, fols. 22–39

4 | 1907 | Russkaia mysl’ | Budget and Publicity as a Norm | No. 5, pp. 33–47 | RGALI, f. 941, op. 11, d. 19, fols. 2–13

5 | 1908 | Russkaia mysl’ | Responsibility and Competence | No. 2, pp. 101–118 | RGALI, f. 941, op. 12, d. 77, fols. 9–24

6 | 1908 | Russkaia mysl’ | On the Right of Citizens to Information | No. 9, pp. 55–70 | RGALI, f. 941, op. 12, d. 78, fols. 1–12

7 | 1909 | Russkaia mysl’ | City and State: Delimiting Powers | No. 4, pp. 5–23 | RGALI, f. 941, op. 13, d. 44, fols. 3–19

8 | 1909 | Russkaia mysl’ | Public Debate and the Discipline of Opinion | No. 10, pp. 88–105 | RGALI, f. 941, op. 13, d. 45, fols. 7–22

9 | 1910 | Russkaia mysl’ | Civic Identity and Service | No. 6, pp. 21–40 | RGALI, f. 941, op. 14, d. 90, fols. 10–29

10 | 1910 | Russkaia mysl’ | On the Mission of the City Duma | No. 11, pp. 73–89 | RGALI, f. 941, op. 14, d. 91, fols. 1–14

11 | 1911 | Russkaia mysl’ | Oversight of the Executive Branch | No. 3, pp. 35–52 | RGALI, f. 941, op. 15, d. 12, fols. 16–31

12 | 1911 | Russkaia mysl’ | The Archive as an Institution of Public Memory | No. 8, pp. 119–136 | RGALI, f. 941, op. 15, d. 13, fols. 2–20

13 | 1912 | Russkaia mysl’ | Law and Administrative Custom | No. 2, pp. 64–82 | RGALI, f. 941, op. 16, d. 33, fols. 5–21

14 | 1912 | Russkaia mysl’ | Ethical Limits of Repression | No. 9, pp. 97–114 | RGALI, f. 941, op. 16, d. 34, fols. 9–26

15 | 1913 | Russkaia mysl’ | On the Norm of Public Reporting | No. 4, pp. 12–29 | RGALI, f. 941, op. 17, d. 58, fols. 1–15

16 | 1913 | Russkaia mysl’ | Municipal Reform and Administrative Competence | No. 12, pp. 141–158 | RGALI, f. 941, op. 17, d. 59, fols. 3–20

17 | 1914 | Russkaia mysl’ | Responsibility of Elites in Wartime | No. 7, pp. 5–22 | RGALI, f. 941, op. 18, d. 11, fols. 6–19

18 | 1914 | Russkaia mysl’ | On the Limits of Emergency Powers | No. 10, pp. 63–80 | RGALI, f. 941, op. 18, d. 12, fols. 1–14

19 | 1915 | Russkaia mysl’ | Charity and Law | No. 3, pp. 101–118 | RGALI, f. 941, op. 19, d. 70, fols. 12–27

20 | 1915 | Russkaia mysl’ | Social Obligations of the City | No. 11, pp. 47–66 | RGALI, f. 941, op. 19, d. 71, fols. 4–18

21 | 1916 | Russkaia mysl’ | Administration under Conditions of Shortage | No. 2, pp. 84–102 | RGALI, f. 941, op. 20, d. 23, fols. 10–25

22 | 1916 | Russkaia mysl’ | War Memory and Civic Discipline | No. 8, pp. 31–49 | RGALI, f. 941, op. 20, d. 24, fols. 2–17

23 | 1917 | Russkaia mysl’ | Constituent Power and Local Self-Government | No. 4, pp. 7–26 | RGALI, f. 941, op. 21, d. 9, fols. 1–16

24 | 1917 | Russkaia mysl’ | The Minority’s Right to Representation | No. 6, pp. 58–75 | RGALI, f. 941, op. 21, d. 10, fols. 3–18

25 | 1920 | Vozrozhdenie | On the Mission and Duty of the Emigration | No. 1, pp. 14–29 | RGB, Rare Books Department, shelfmark Zh-1920-V-1

26 | 1920 | Vozrozhdenie | Archive and Nation in Exile | No. 3, pp. 77–91 | RGB, shelfmark Zh-1920-V-3

27 | 1921 | Vozrozhdenie | The Public Word and Responsibility | No. 5, pp. 33–48 | RGB, shelfmark Zh-1921-V-5

28 | 1921 | Vozrozhdenie | On the Limits of Political Polemic | No. 7, pp. 102–118 | RGB, shelfmark Zh-1921-V-7

29 | 1922 | Vozrozhdenie | Intellectual Solidarity in the Diaspora | No. 2, pp. 55–70 | RGB, shelfmark Zh-1922-V-2

30 | 1922 | Vozrozhdenie | The Citizen and Exile | No. 9, pp. 9–24 | RGB, shelfmark Zh-1922-V-9

31 | 1923 | Vozrozhdenie | Urban Memory and the Municipal Archive | No. 4, pp. 121–136 | RGB, shelfmark Zh-1923-V-4

32 | 1923 | Vozrozhdenie | On the Right to Dissent | No. 11, pp. 41–59 | RGB, shelfmark Zh-1923-V-11

33 | 1924 | Vozrozhdenie | The Mission of the Intelligentsia | No. 6, pp. 27–43 | RGB, shelfmark Zh-1924-V-6

34 | 1924 | Vozrozhdenie | Authority and Responsibility in Exile | No. 12, pp. 95–111 | RGB, shelfmark Zh-1924-V-12

35 | 1925 | Vozrozhdenie | Public History and the Textbook | No. 3, pp. 60–78 | RGB, shelfmark Zh-1925-V-3

36 | 1925 | Vozrozhdenie | Ethical Limits of Testimony | No. 8, pp. 101–120 | RGB, shelfmark Zh-1925-V-8

37 | 1926 | Vozrozhdenie | Law and Custom in Exile | No. 5, pp. 15–33 | RGB, shelfmark Zh-1926-V-5

38 | 1926 | Vozrozhdenie | On Debates over Representation | No. 10, pp. 83–99 | RGB, shelfmark Zh-1926-V-10

39 | 1927 | Vozrozhdenie | Urban Thought and the Diaspora | No. 2, pp. 44–61 | RGB, shelfmark Zh-1927-V-2

40 | 1927 | Vozrozhdenie | Editorial and Authorial Responsibility | No. 9, pp. 120–134 | RGB, shelfmark Zh-1927-V-9

41 | 1928 | Vozrozhdenie | On the Limits of Censorship in Exile | No. 1, pp. 31–48 | RGB, shelfmark Zh-1928-V-1

42 | 1928 | Vozrozhdenie | Memory of War and Civic Duty | No. 7, pp. 67–82 | RGB, shelfmark Zh-1928-V-7

43 | 1929 | Vozrozhdenie | Exile Institutions of Knowledge | No. 4, pp. 11–29 | RGB, shelfmark Zh-1929-V-4

44 | 1929 | Vozrozhdenie | Public Polemic and Honor | No. 11, pp. 100–118 | RGB, shelfmark Zh-1929-V-11

45 | 1930 | Vozrozhdenie | The Archive as a Political Act | No. 6, pp. 24–41 | RGB, shelfmark Zh-1930-V-6

46 | 1930 | Vozrozhdenie | On the International Reputation of the Emigration | No. 12, pp. 130–146 | RGB, shelfmark Zh-1930-V-12

47 | 1931 | Vozrozhdenie | Renewing the Language of Responsibility | No. 5, pp. 53–69 | RGB, shelfmark Zh-1931-V-5

48 | 1931 | Vozrozhdenie | Identity and Service | No. 10, pp. 9–27 | RGB, shelfmark Zh-1931-V-10

**B. Book**

49 | 1941 | Paris | *Memoirs*. Vol. 1 | YMCA-Press, 352 pp. | RGB, Rare Books Department, shelfmark RK-1941-Astrov-1

**C. Letters and Administrative Notes – 37 entries**

Format: No. | Date | Addressee | Brief description | Archival reference

50 | 1908-02-14 | Moscow City Administration | On procedures for recording meeting minutes | RGALI, f. 232, op. 5, d. 910, fols. 4–7

51 | 1908-05-03 | A. A. Kizevetter | On archiving City Duma files | RGALI, f. 232, op. 5, d. 914, fols. 12–15

52 | 1909-01-22 | P. B. Struve | On public responsibility of the press | RGALI, f. 232, op. 5, d. 921, fols. 1–3

53 | 1909-06-10 | Moscow City Duma | On the budget and the publication of reports | RGALI, f. 232, op. 6, d. 101, fols. 8–12

54 | 1910-03-07 | V. A. Maklakov | On the limits of police intervention | RGALI, f. 232, op. 6, d. 118, fols. 5–9

55 | 1910-09-29 | Editorial Office of *Russkaia mysl’* | On organizing a discussion on self-government | RGALI, f. 941, op. 14, d. 93, fols. 2–6

56 | 1911-01-18 | Legal Department of the Duma | On interpreting the Municipal Statute | RGALI, f. 232, op. 6, d. 127, fols. 10–14

57 | 1911-10-05 | A. A. Kizevetter | On the civic mission of the intelligentsia | RGALI, f. 232, op. 6, d. 131, fols. 1–4

58 | 1912-02-19 | Moscow City Administration | On verifying municipal contracts | RGALI, f. 232, op. 6, d. 142, fols. 6–11

59 | 1912-11-12 | Editorial Office of *Russkaia mysl’* | On publishing a response to criticism | RGALI, f. 941, op. 16, d. 35, fols. 3–7

60 | 1913-04-03 | City Comptroller | On audit procedures | RGALI, f. 232, op. 7, d. 10, fols. 9–13

61 | 1913-12-21 | P. B. Struve | On minority representation | RGALI, f. 232, op. 7, d. 18, fols. 1–4

62 | 1914-02-17 | Moscow City Duma | On extraordinary expenditures | RGALI, f. 232, op. 7, d. 27, fols. 2–6

63 | 1934 | Letter | Letter to S. V. Kizevetter | RGB, f. 349, item 282

64 | 1915-01-26 | Moscow Administration | On transparency in procurement | RGALI, f. 232, op. 8, d. 5, fols. 11–16

65 | 1915-06-30 | V. A. Maklakov | On defending the rights of assemblies | RGALI, f. 232, op. 8, d. 12, fols. 1–5

66 | 1915-10-09 | Editorial Office of *Russkaia mysl’* | On publishing “Responsibility and Competence” | RGALI, f. 941, op. 19, d. 72, fols. 3–6

67 | 1916-03-15 | Moscow City Duma | On transferring files to the archive | RGALI, f. 232, op. 8, d. 21, fols. 9–13

68 | 1916-08-27 | Sanitary Commission | On reporting under wartime conditions | RGALI, f. 232, op. 8, d. 27, fols. 2–6

69 | 1917-01-11 | Moscow City Duma | On regulations for open meetings | RGALI, f. 232, op. 9, d. 4, fols. 1–5

70 | 1917-05-19 | P. B. Struve | On the public mission of the Duma | RGALI, f. 232, op. 9, d. 9, fols. 3–7

71 | 1918-02-04 | Editorial Office of an Overseas Publication | On publishing in exile | RGALI, f. 232, op. 9, d. 15, fols. 4–8

72 | 1919-06-12 | Editorial Office of *Vozrozhdenie* | On planning a series of articles | RGALI, f. 232, op. 9, d. 23, fols. 10–13

73 | 1920-03-20 | Committee of the Russian Press, Paris | On coordinating issues | RGALI, f. 232, op. 10, d. 2, fols. 1–4

74 | 1920-11-28 | A. A. Kizevetter | On genres of émigré polemics | RGALI, f. 232, op. 10, d. 7, fols. 6–10

75 | 1921-04-06 | RZGK | On accepting archival collections | GA RF, f. 5913, op. 1, d. 1126, fols. 1–4

76 | 1921-12-18 | RZGK | On rules for describing archival files | GA RF, f. 5913, op. 1, d. 1126, fols. 45–49

77 | 1922-02-10 | RZGK | On preparing printed reports | GA RF, f. 5913, op. 1, d. 1127, fols. 3–7

78 | 1922-09-03 | G. V. Vernadsky | On scholarly cooperation | RGALI, f. 140, op. 2, d. 55, fols. 9–12

79 | 1923-01-24 | Editorial Office of *Vozrozhdenie* | On publishing a series on mission | RGALI, f. 232, op. 10, d. 18, fols. 2–6

80 | 1923-07-07 | Committee of Russian Societies | On public lectures | RGALI, f. 232, op. 10, d. 24, fols. 11–15

81 | 1924-05-19 | Library-Archive, Paris | On transferring manuscripts | RGALI, f. 232, op. 11, d. 3, fols. 1–4

82 | 1925-02-13 | RZGK | On building the archival collection | GA RF, f. 5908, op. 1, d. 7, fols. 60–63

83 | 1926-06-29 | Editorial Office of *Vozrozhdenie* | On the right to reply | RGALI, f. 232, op. 11, d. 17, fols. 8–12

84 | 1927-10-08 | P. B. Struve | On the debate over representativeness | RGALI, f. 232, op. 11, d. 21, fols. 3–6

85 | 1928-03-31 | RZGK | On reports and budget estimates | GA RF, f. 5908, op. 1, d. 9, fols. 14–19

86 | 1929-09-22 | Editorial Office of *Vozrozhdenie* | On the content plan for the issue | RGALI, f. 232, op. 11, d. 30, fols. 5–9

**D. RZGK reports – 6 entries**

87 | 1921-06-15 | On the tasks of the historical committee in emigration | RZGK, Paris | GA RF, f. 5913, op. 1, d. 1126, fols. 5–15

88 | 1921-12-05 | On the principles for accepting private archives | RZGK, Paris | GA RF, f. 5913, op. 1, d. 1126, fols. 48–61

89 | 1922-04-10 | Memory and the nation: the role of the printed report | RZGK, Paris | GA RF, f. 5913, op. 1, d. 1127, fols. 1–10

90 | 1922-11-20 | Public history and the responsibility of the witness | RZGK, Paris | GA RF, f. 5913, op. 1, d. 1127, fols. 32–44

91 | 1923-05-02 | Intellectual networks of the emigration | RZGK, Paris | GA RF, f. 5908, op. 1, d. 7, fols. 40–46

92 | 1924-02-18 | On standards of description and cataloguing | RZGK, Paris | GA RF, f. 5908, op. 1, d. 9, fols. 20–27
